# Image en popcorn cérébrale

**DOI:** 10.11604/pamj.2016.23.3.8583

**Published:** 2016-01-08

**Authors:** Rachid Ammor, Assou Ajja

**Affiliations:** 1 Hôpital Militaire My Ismael, Meknès, Maroc

**Keywords:** Crises convulsives généralisées, IRM cérébrale, image en pop corn, traitement antiépileptique

## Image en médecine

Les cavernomes cérébraux sont des lésions vasculaires définies par la présence de capillaires malformés sans interposition de tissu nerveux. Ce sont des lésions relativement rares (0,1à 0,5% de la population générale dont moins de 5% sont symptomatiques), la symptomatologie clinique est dominée par l'hémorragie et l’épilepsie et dépend aussi de la localisation. L'IRM cérébrale est l'examen de référence pour le diagnostic, l'approche thérapeutique et le suivi. Nous rapportons ici l'observation clinique d'un jeune homme de 27 ans, sans antécédents, qui a présenté deux crises convulsives généralisées à une semaine d'intervalle. L'examen clinique a été normal. L'IRM cérébrale a objectivé une lésion frontale gauche avec aspect typique en pop corn ou poivre et sel associant hyper signal (saignement récent ou calcifications) et hypo signal (dépôt d'hémosidérine=saignement ancien) sur les séquences pondérées T2. Le patient a été mis sous traitement antiépileptique a base d'acide valproïque avec contrôle total des crises.

**Figure 1:**
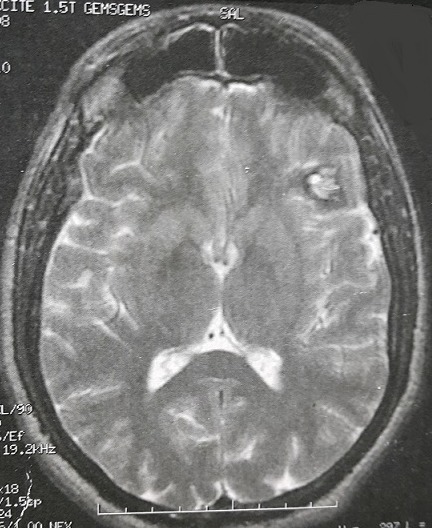
IRM cérébrale en coupe axiale séquence pondérée T2, montrant une lésion frontale gauche en hyper et hypo signal de 2,2/2,5cm

